# Supplementary material for: Estimating the duration of antibody positivity and likely time of Leptospira infection using data from a cross-sectional serological study in Fiji
Source: PLoS Negl Trop Dis. 2022 Jun 13;16(6):e0010506. doi: 10.1371/journal.pntd.0010506 (PMC9232128; doi:10.1371/journal.pntd.0010506)
Supplement: S1 Table — (PDF) [file pntd.0010506.s001.pdf]

S1 Table. Summary, advantages and disadvantages of MAT and ELISA test used for the diagnosis of leptospirosis [1-5].

Diagnostic test	Summary	Advantage	Disadvantage
MAT	Patient serum is incubated with live antigen leptospires. Agglutination then occurs, which is detected using dark-field microscopy. Live antige leptospires are diluted sequentially, and the highest dilution in which 50% agglutination occurs is recorded. IgG and IgM antibodies can be detected using dark field microscopy.	'Gold standard' test, due to high specificity and ability to distinguish between serovars.	Requires maintenance of a panel of live leptospires, it can be time consuming and difficult to interpret the results and requires the correct selection of leptospire serovars on the panel. Cross reaction between different serogroups may occur.
ELISA	Detection of (usually) IgM antibodies in patient serum using a broad- spectrum antigen against pathogenic *Leptospira* spp.	More sensitive than MAT during the acute phase of the illness. It is easy to perform and results are rapidly available.	Not serovar-specific and detects both pathogenic and non-pathogenic *Leptospira* spp. Sensitivity and specificity variable.

MAT, microscopic agglutination test; ELISA, enzyme-linked immunosorbent assay.

References:

1. Picardeau M. Diagnosis and epidemiology of leptospirosis. Médecine et Maladies Infectieuses 2013;43:1–9. doi:10.1016/j.medmal.2012.11.005
2. Picardeau M, Bertherat E, Jancloes M, et al. Rapid tests for diagnosis of leptospirosis Current tools and emerging technologies. Diagnostic Microbiology and Infectious Disease 2014;78:1–8. doi:10.1016/j.diagmicrobio.2013.09.012
3. Haake DA, Levett PN. Leptospirosis in humans. *Current topics in microbiology and immunology* 2015;**387**:65–97. doi:10.1007/978-3-662-45059-8_5
4. Levett PN. Leptospirosis. *Clinical Microbiology Reviews* 2001;**14**:296–326. doi:10.1128/CMR.14.2.296-326.2001
5. Musso D, La Scola B. Laboratory diagnosis of leptospirosis: A challenge. *Journal of Microbiology, Immunology and Infection* 2013;**46**:245–52. doi:10.1016/j.jmii.2013.03.001
