# Supplementary material for: Estimating the duration of antibody positivity and likely time of Leptospira infection using data from a cross-sectional serological study in Fiji
Source: PLoS Negl Trop Dis. 2022 Jun 13;16(6):e0010506. doi: 10.1371/journal.pntd.0010506 (PMC9232128; doi:10.1371/journal.pntd.0010506)
Supplement: S2 Table — (PDF) [file pntd.0010506.s002.pdf]

S2 Table. Description of the different models fitted and priors used.

Model	Priors	Number of parameters
Catalytic model	FOI ~ Uniform(0,0.5)	1
Reverse catalytic model	FOI ~ Uniform(0,0.5)<br>Waning ~ Uniform(0,10)	2
Reverse catalytic model by sex	FOI ~ Uniform(0,0.5)<br>Waning ~ Uniform(0,10)	3
Reverse catalytic model by administrative division	FOI ~ Uniform(0,0.5)<br>Waning ~ Uniform(0,10)	4
Reverse catalytic model by serovar	FOI ~ Uniform(0,0.1)<br>Waning ~ Uniform(0,10)	5
Constant FOI with 1 outbreak (2 years)	FOI ~ Uniform(0,0.1)<br>Waning ~ Uniform(0,10)<br>T1 ~ Uniform(0,2)	3
Constant FOI with 1 outbreak (5 years)	FOI ~ Uniform(0,0.1)<br>Waning ~ Uniform(0,10)<br>T1 ~ Uniform(0,5)	3
No constant FOI & 1 Outbreak (10 years)	FOI ~ Uniform(0,0.1)<br>Waning ~ Uniform(0,10)<br>T1 ~ Uniform(0,10)	3

FOI, Force of infection; T1, timing of the outbreak.
