# Supplementary material for: Estimating the duration of antibody positivity and likely time of Leptospira infection using data from a cross-sectional serological study in Fiji
Source: PLoS Negl Trop Dis. 2022 Jun 13;16(6):e0010506. doi: 10.1371/journal.pntd.0010506 (PMC9232128; doi:10.1371/journal.pntd.0010506)
Supplement: S3 Table — Estimating the FOI and waning from a high FOI and low FOI setting. (PDF) [file pntd.0010506.s003.pdf]

S3 Table. Simulation recovery study. Estimating the FOI and waning from a high FOI and low FOI setting.

Setting	True parameter values		Model estimates	
	FOI	Waning	FOI (95% CrI)	Waning (95% CrI)
High FOI	0.05	0.1	0.051 (0.039 - 0.068)	0.101 (0.074 - 0.138)
Low FOI	0.005	0.1	0.032 (0.004 - 0.178)	0.715 (0.072 – 4.028)

FOI, Force of infection
