# Supplementary material for: Estimating the duration of antibody positivity and likely time of Leptospira infection using data from a cross-sectional serological study in Fiji
Source: PLoS Negl Trop Dis. 2022 Jun 13;16(6):e0010506. doi: 10.1371/journal.pntd.0010506 (PMC9232128; doi:10.1371/journal.pntd.0010506)
Supplement: S4 Table — Antibody drop time was defined as the time taken in months for antibodies to drop one antibody titre level (e.g. from 1:100 to 1:50). (PDF) [file pntd.0010506.s004.pdf]

S4 Table. Results from the mixed-effects linear model from the point source outbreak in Italy (Lupidi *et al.*). Antibody drop time was defined as the time taken in months for antibodies to drop one antibody titre level (e.g. from 1:100 to 1:50).

Serovar	Antibody titre drop time in months (95% CrI)	Time taken to reach undetectable levels (years)
Bratislava	6.94 (5.63 - 9.05)	6.05
Australis	9.30 (6.88 - 15.08)	6.66
Iora	7.51 (6.38- 9.12)	6.99

CrI, credible interval.
