# Supplementary figures and images for: Estimating the duration of antibody positivity and likely time of Leptospira infection using data from a cross-sectional serological study in Fiji
Source: PLoS Negl Trop Dis. 2022 Jun 13;16(6):e0010506. doi: 10.1371/journal.pntd.0010506 (PMC9232128; doi:10.1371/journal.pntd.0010506)

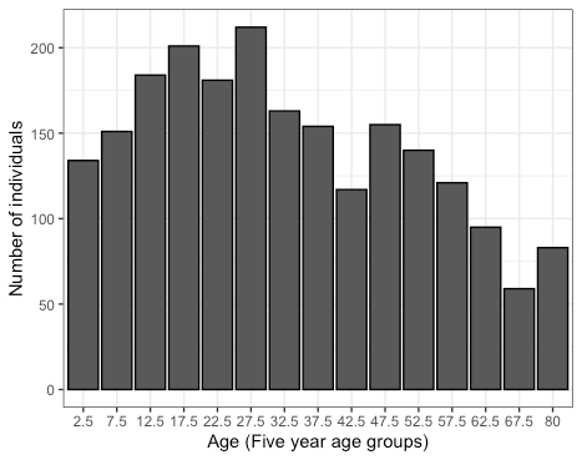

Supplement: S1 Fig — (TIFF) [file pntd.0010506.s005.tiff]

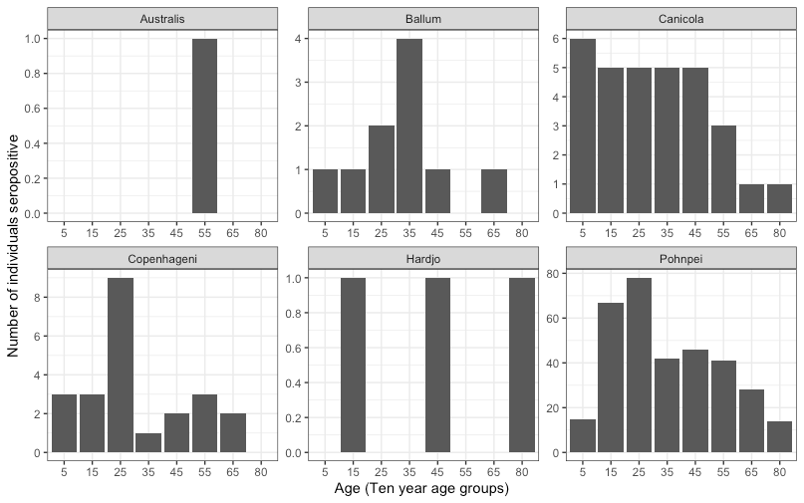

Supplement: S2 Fig — Individuals that had the same titre for two serovars, and therefore infecting titre could not be assumed, were excluded (n = 18). (TIFF) [file pntd.0010506.s006.tiff]

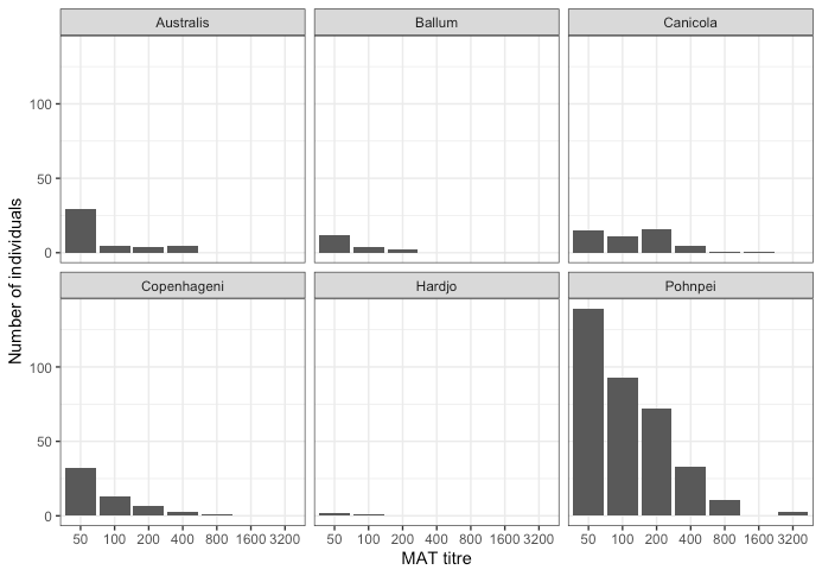

Supplement: S3 Fig — 89 individuals had titres for more than one serovar, and so are included more than once in this plot (n = 520). (TIFF) [file pntd.0010506.s007.tiff]

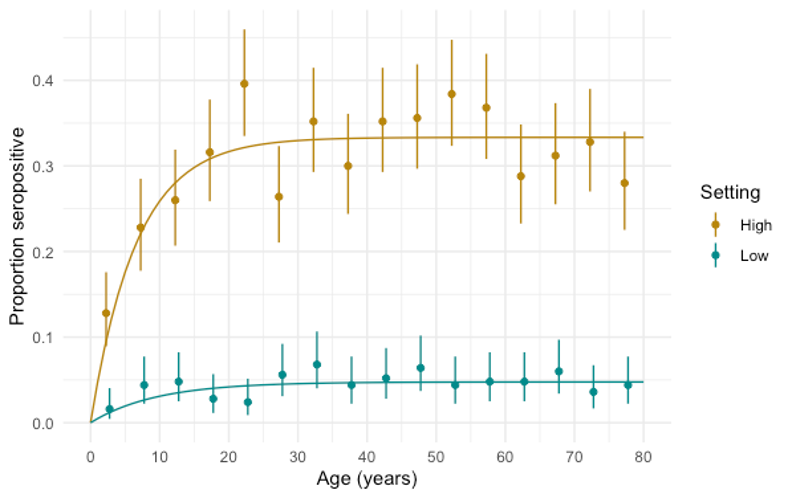

Supplement: S4 Fig — Sample estimates (mean and 95% binomial confidence interval) and model fit (solid line) for the high FOI (shown in orange) and low FOI (shown in blue) scenario. Under the high FOI scenario, the parameter estimates obtained were similar to the true parameter values. Under the low FOI scenario, the model was able to reproduce the data, but there was much greater uncertainty in the true underlying parameters. (TIFF) [file pntd.0010506.s008.tiff]

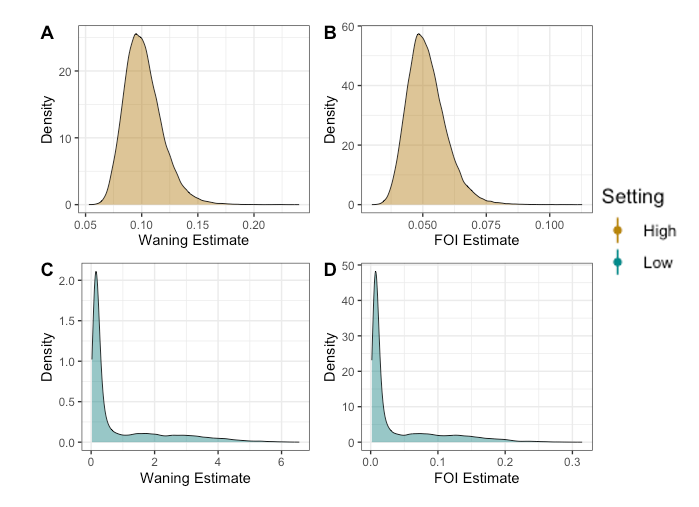

Supplement: S5 Fig — Posterior distributions for waning and force of infection (FOI) for the high FOI scenario (orange) and low FOI scenario (blue). Under the high FOI scenario, the parameter estimates obtained were similar to the true parameter values. Under the low FOI scenario, although the true parameter values were included within the 95% credible intervals, there was much greater uncertainty in the estimates. (TIFF) [file pntd.0010506.s009.tiff]

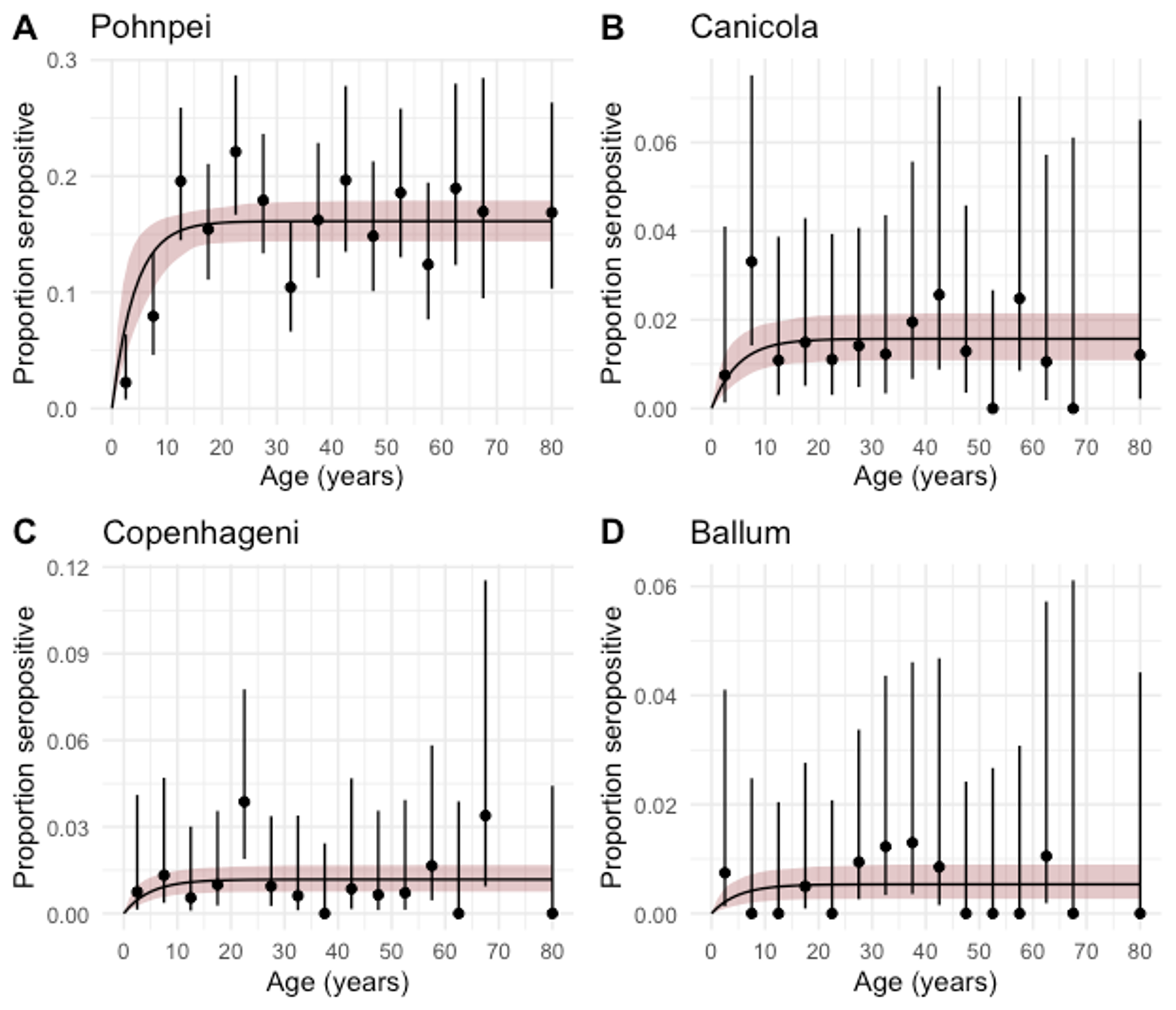

Supplement: S6 Fig — Proportion of seropositive individuals by age (black points represent the mean and the error bars represent the binomial 95% confidence intervals), from national serosurvey conducted in Fiji in 2013 (n = 2,152) by serovar Pohnpei (A), Canciola (B), Copenhageni (C) and Ballum (D). The reverse catalytic model is shown for each serovar including model 95% credible intervals (red shading). (TIFF) [file pntd.0010506.s010.tiff]

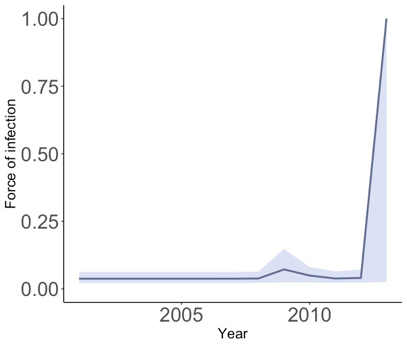

Supplement: S7 Fig — (TIFF) [file pntd.0010506.s011.tiff]

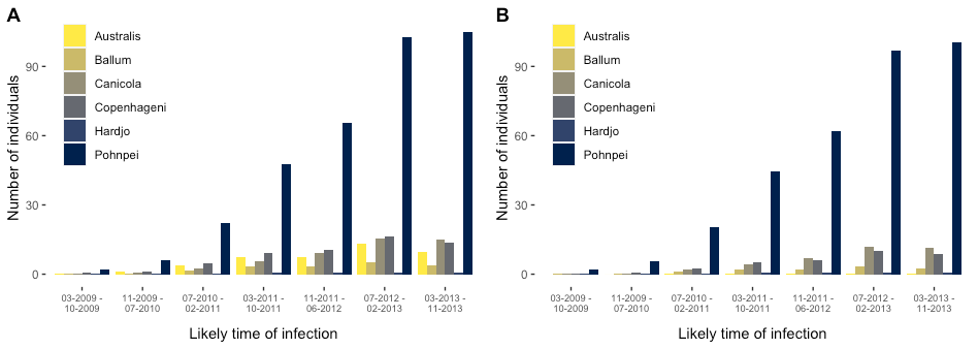

Supplement: S8 Fig — (A) assumes that individuals can be seropositive for more than one serovar at different times (n = 520), whilst (B) using results of the serovar associated with the highest titre (n = 417). (TIFF) [file pntd.0010506.s012.tiff]

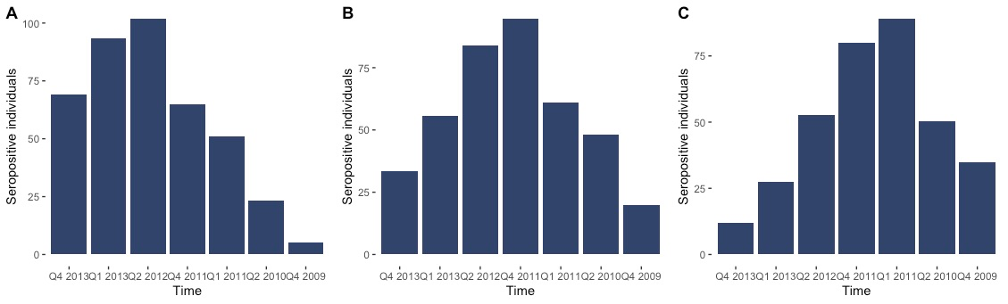

Supplement: S9 Fig — The initial titre distributions were shifted to correspond to a geometric mean (a) one dilution titre higher, (b) two dilutions titres higher and (c) three dilution titres higher. (TIFF) [file pntd.0010506.s013.tiff]
