# Supplementary material for: Estimating the duration of antibody positivity and likely time of Leptospira infection using data from a cross-sectional serological study in Fiji
Source: PLoS Negl Trop Dis. 2022 Jun 13;16(6):e0010506. doi: 10.1371/journal.pntd.0010506 (PMC9232128; doi:10.1371/journal.pntd.0010506)
Supplement: Attachment — Submitted filename: responseLetter_REES.docx [file pntd.0010506.s014.docx]

Eleanor Rees

Department of Infectious Disease Epidemiology

London School of Hygiene & Tropical Medicine,

Keppel Street,

London WC1E 7HT

Eleanor.rees1@lshtm.ac.uk

6^th^ April 2022

**Re:** **Estimating the duration of antibody positivity and likely time of *Leptospira* infection using data from a cross-sectional serological study in Fiji**

Dear Editors,

We would like to thank the reviewers for their comments on the above manuscript; our proposed amendments and responses are laid out below.

We have changed the manuscript significantly to address the reviewers’ comments and believe that it has made a substantial improvement.

Reviewer #1

Overall, I thought the paper was great!

\# Introduction

The introduction is very well written.

\# Methods

I think a brief phrase on the rationale for the selection of these serovars would be useful. The authors reference another paper, but just a quick summary so readers don't have to jump into the other reference might be nice.

Thank you for taking the time to review our article. Thank you for the suggestion, we have included further details of the serovar selection within the methods:

*“The presence of anti-Leptospira antibodies in sera collected from participants was determined using the MAT with a panel of six serovars, Leptospira interrogans serovars Pohnpei (serogroup Australis), Australis (serogroup Australis), Canicola (serogroup Canicola), Copenhageni (serogroup Icterohaemorrhagiae), Hardjo (serogroup Sejroe), and Leptospira borgpetersenii serovar Ballum (serogroup Ballum). An initial panel of 21 pathogenic serovars was used on a random selection of ~10% of the total samples. In addition, this 21 serovar panel was used on 199 Leptospira ELISA-positive samples collected from patients with suspected clinical leptospirosis in Fiji in 2012 and 2013. The serogroups most commonly detected in the clinical and serosurvey samples were then chosen and included in the final panel of six serovars. Further details on selection of the serovars for the MAT panel have been previously described by Lau et al. [29]. Samples were tested at titre dilutions from 1:50 to 1:3200, and MAT titres of ≥1:50 were defined as seropositive. A higher antibody titre dilution is usually considered indicative of a more recent infection (i.e. MAT ≥1:400), whilst a lower antibody titre of a past infection. MATs were conducted at the WHO Collaborating Centre for Reference and Research on Leptospirosis in Brisbane, Australia.”*

For the reverse catalytic models that were serovar specific, was the rate of waning estimated independently for each? It appears in the results that waning was assumed to be the same across the serovars? It would be interesting to see if these emerge consistently in the separate models. In some ways, I think that these results should really be analyzed in a serovar specific way, given the biology of lepto (although the authors themselves discuss the limitations of the mat given cross-reactivity and also fuzziness biologically of serovars). The authors note that they use a narrower prior on the FOI for the serovar specific analyses, which maybe indicates that these data are not robust enough for serovar specific estimates of foi and waning, which I think is totally fine, but it would be good to present these results at least in the supplementary materials and state so up front. I think this is less of an issue as the subsequent analyses at the individual level largely help with this limitation.

The rate of waning was assumed to be constant across all serovars. This could have been a little clearer, so we have clarified this in the methods:

*“For the analysis by serovar, the FOI was allowed to vary by serovar, whilst waning was held constant across serovars.”*

In a setting where the FOI is high, the FOI and waning are easier to estimate. When FOI is lower, there are fewer infection events over time and hence greater uncertainty, and the FOI and waning can be more challenging to estimate. For this reason we chose to keep waning constant, and estimate only the FOI by serovar.

To highlight the challenges with using the reverse catalytic model in a low infection setting, we have included a simulation study. We simulated data in a high and low FOI setting and then attempted to recover the parameters. Using the reverse catalytic model we generated two models, a high FOI model (FOI, 0.05 and waning 0.1) and low FOI model (FOI 0.005 and waning 0.1). We then sampled 50 times from each 5-year age group using a binomial distribution to generate seropositive and seronegative individuals. We then re-fitted a reverse catalytic model to both datasets to estimate the FOI and waning in both settings.

In the high FOI setting we were able to get similar estimates for both FOI and waning, with the true parameter values included within the 95% credible intervals. However, in the low FOI setting, although the true parameter values were included within the 95% credible intervals, there was much greater uncertainty in the parameter estimates

**Supplementary Table 3**. Simulation recovery study. Estimating the FOI and waning from a high FOI and low FOI setting

| Setting | True parameter values | | Model estimates | |
| --- | --- | --- | --- | --- |
|  | FOI | Waning | FOI (95% CrI) | Waning (95% CrI) |
| High FOI | 0.05 | 0.1 | 0.051 (0.039 - 0.068) | 0.101 (0.074 - 0.138) |
| Low FOI | 0.005 | 0.1 | 0.032 (0.004 - 0.178) | 0.715 (0.072 – 4.028) |

**Supplementary Figure 4**. Simulation recovery study. Sample estimates (mean and 95% binomial confidence interval) and model fit (solid line) for the high FOI (shown in orange) and low FOI (shown in blue) scenario. Under the high FOI scenario, the parameter estimates obtained were similar to the true parameter values. Under the low FOI scenario, the model was able to reproduce the data, but there was much greater uncertainty in the true underlying parameters.

**Supplementary Figure 5**. Simulation recovery study. Posterior distributions for waning and force of infection (FOI) for the high FOI scenario (orange) and low FOI scenario (blue). Under the high FOI scenario, the parameter estimates obtained were similar to the true parameter values. Under the low FOI scenario, although the true parameter values were included within the 95% credible intervals, there was much greater uncertainty in the estimates.

This simulation study has now been included in the methods:

*“Waning was held constant across serovars as when the FOI is lower, FOI and waning can be more challenging to estimate. This is because there are fewer infection events over time and hence greater uncertainty. To highlight this, we did a simulation recovery study where we recovered the FOI and waning estimates from two settings, a high FOI and low FOI setting. Using the reverse catalytic model we generated two models, a high FOI model (FOI, 0.05 and waning 0.1) and low FOI model (FOI 0.005 and waning 0.1). We then sampled 50 times from each 5-year age group using a binomial distribution to generate seropositive and seronegative individuals (Supplementary Fig.4). We then re-fitted a reverse catalytic model to both datasets to estimate the FOI and waning in both settings. In the high FOI setting we were able to get similar estimates for both FOI and waning, with the input parameter estimates included within the 95% credible intervals. However, in the low FOI setting, although the true parameter values were included within the 95% credible intervals, there was much greater uncertainty in the parameter estimates (Supplementary Table 3 and Supplementary Fig. 5).”*

The link to the github repository was broken?

Apologies - you should now be able to access it.

#Results

A couple of visualization questions/suggestions:

In figure 3, how are the raw data being presented? Are the black dots with the error bars proportion of indivduals that are seropositive and the binomial uncertainty around that proportion?

Yes, the black dots are the mean, and the error bars are the 95% binomial confidence intervals. We have now updated the figure legend to include this information.

*“Figure 3. Proportion of seropositive individuals by age (black points represent the mean and the error bars represent the binomial 95% confidence intervals), from national serosurvey conducted in Fiji in 2013 (n = 2,152). The catalytic model is shown in red (A) and reverse catalytic model is shown in blue (B), including model 95% credible intervals (darker shading) and the sampling uncertainty (binomial, lighter shading).*”

How wide are the age bins? It would be nice to know how many total individuals are in each of those bins. A supplementary figure showing the age distribution would be nice and also the age distribution of seropositives by each serovar. It would also be nice to see the same type of figure in Fig 3B for each of the serotypes.

Thank you for the suggestions for additional plots. We have added a supplementary figure showing the age distribution of individuals included within the seroprevalence survey by five year age groups (Supplementary Figure 1). This grouped dataset is also provided on the github repository as a csv. We have also added a plot showing the age distribution of individuals by serovar (Supplementary Figure 2).

**Supplementary Figure 1**. Number of individuals included within the 2013 leptospirosis serosurvey by five-year age groups.

**Supplementary Figure 2**. Number of individuals seropositive by serovar by ten-year age groups (n = 399). Individuals that had the same titre for two serovars, and therefore infecting titre could not be assumed, were excluded (n=18).

Finally, we have also included a figure showing the reverse catalytic model by serovar (Supplementary Fig. 6).

**Supplementary Figure 6**. Proportion of seropositive individuals by age (black points represent the mean and the error bars represent the binomial 95% confidence intervals), from national serosurvey conducted in Fiji in 2013 (n = 2,152) by serovar Pohnpei (A), Canciola (B), Copenhageni (C) and Ballum (D). The reverse catalytic model is shown for each serovar including model 95% credible intervals (red shading).

For figure 4 and it's counterparts in the supplement, a couple of suggestions:

- Reverse the axis so that it goes from earliest to latest (or in this case the time of sampling). I think this is a bit more intuitive for the reader.

- I personally find stacked bar graphs really hard to see. Could the serovars be split into separate panels? Or density plots if there are enough data?

Thank you for the suggestion - We have reversed the x axis of figure 4 to make it more intuitive to read.

The purpose of this figure was to demonstrate the total number of infections occurring in each year, and a break-down by serovar would not allow for that to be observed as easily. Instead, we have included a non-stacked plot by serovar in the supplementary material (Supplementary Figure 8).

**Supplementary Figure 8**. Estimating the most likely time of infection from leptospirosis seroprevalence data from Fiji by serovar. (A) assumes that individuals can be seropositive for more than one serovar at different times (n = 520), whilst (B) using results of the serovar associated with the highest titre (n = 417).

It would also be nice to see how much uncertainty there is for each individual estimate of timing of infection (maybe just an x-y plot with titre on x and jittered points showing each individual estimate of the time from initial infection with uncertainty on the y) for each serovar? I may be misinterpreting the output of the model, but this seems important for evaluating whether this individual method works.

Due to the uncertainty in individual-level titre trajectories, the timing of infection was reported as an expectation at the population level, rather than the individual level infection histories. For example, suppose 100 individuals all have a 50% probability of being infected in year A or year B; at the individual level, infection timing can’t be reported with confidence, but the expectation of 50 infections in each year can be.

Using population-level titre distribution from the 2013 Fiji seroprevalence survey, we compared these values to the population titre distribution of 2012-2013 suspected clinical leptospirosis cases, to determine what the distribution of initial titres may have been. We then used the antibody decay rate estimated from *Lupidi* et al. to transform this into when infection may have occurred, and summed over the individual. This was repeated for each titre level.

This could have been clearer so we have added to our methods:

*“Using the MAT antibody titres from the 2013 Fiji seroprevalence survey, we estimated the timing of infection of participants. Due to uncertainty associated with individual titre estimates – and hence timings – the dynamics of infection was aggregated and reported as the population level expectation.”*

*“To reconstruct the timing of infection from the 2013 seroprevalence survey we combined the 2012 Fiji clinically suspected cases with the estimated rate at which individual responses wane by one antibody dilution titre. First, using the 2012 Fiji clinically suspected cases (n = 199), we estimated the geometric mean antibody titre from the MAT-positive cases (n = 66). We then used the MAT antibody distribution of the 2012 Fiji clinically suspected cases, combined with the antibody decay estimates from Lupidi et al. [23], to analyse the 2013 seroprevalence data. For each titre level from the 2013 seroprevalence survey, the possible initial titre levels were estimated, based on the proportions from the 2012 Fiji clinically suspected case distribution results. Using the rate of decline of antibody titres, the initial titre levels were transformed into an estimated time since infection, reconstructing the potential timing of infection at the population level. An example for one titre is shown in Fig. 2. As individuals could be seropositive for more than one serovar, two separate analyses were conducted, one where infections with different serovars were assumed to be independent events (n = 520), and one where only the highest titre was used (n = 417).”*

\# Discussion

Very nice discussion, the authors discuss many of the challenges in analyzing lepto data that are relevant to understanding their results. A bit on the specific serotypes in this study and the host biology would be nice (unless I just missed it).

Thank you for your comments. We have added more details regarding host-pathogen in the introduction:

*“Certain serovars are more commonly associated with particular hosts, for example Leptospira interrogans serovar Hardjo is frequently associated with cattle, and Leptospira interrogans serovar Canicola with dogs [16,17]. However, these associations are not absolute, and there is considerable heterogeneity in the dominant serovars in both animals and humans each country, even in remote islands [3]. ”*

As well as expanding the serovar-specific results in the discussion:

*“A previous study describing the human serosurvey in Fiji found that there were differences in serovar distribution by age and location, suggesting that there are different risk factors of disease transmission between sub-groups [29]. For example, livestock could be more important drivers in rural areas, with rodents being more important in urban areas. However, in this setting there was one dominant serovar (serovar Pohnpei), limiting the ability to identify serovar-specific risk factors.”*

Reviewer #2

I think this is a great paper. It brings together an innovative mix of methods (surveys, assays, statistical and mathematical models) to address important and understudied questions related to the epidemiology of leptospirosis.

**Major comments**

My major comment is that there is a potential contradiction between how the results from the applied approaches are interpreted. The reversible catalytic model makes an assumption of historically constant transmission, and was used to provide two of the key estimates: the duration of antibody persistence = 8.33 (4.76, 12.5) years; and the annual attack rate = 3.15% (2.18%, 5.16%). In contrast, two different approaches: (i) time-dependent sero-catalytic models; and (ii) longitudinal antibody kinetic analysis suggest that the majority of transmission was recent.

The authors should weight the evidence for the two competing scenarios (constant transmission or recent outbreak), and then highlight the results from the scenario with best evidence. Retaining the other estimates as supplementary information.

Thank you for taking the time to review our article. We appreciate that we could have been clearer about our focus here. In the first part of our analysis, we compared the reverse catalytic constant FOI model with the reverse catalytic time-varying FOI model, using WAIC for model comparison. There was very little difference in WAIC between both models, therefore we were not able to distinguish which model had the most support. However, the results of antibody persistence – which was our key biological parameter of interest – were very similar for both models, indicating that this was robust to model structure. Given this, we have updated our discussion and abstract to focus on the findings around antibody persistence from both models, and subsequent application to the individual antibody kinetic data, rather than focussing on the non-identifiable conclusions about timing in the reverse catalytic model:

*Discussion:*

*“Using the reverse catalytic model we also estimated the persistence of detectable anti-Leptospira antibodies to be 8.33 years (4.76 - 12.50 years). Similar estimates were obtained when analysed by sex, administrative division and serovar. Furthermore, since large seasonal outbreaks of leptospirosis are known to occur in Fiji, we explored how a time-varying FOI influenced our estimates of the duration of seropositivity, and found that our estimates remained similar [7.25 years (3.36 – 11.36), for the constant FOI with one outbreak in the last five years]. There was little difference between the WAIC estimates of the reverse catalytic model and the time-varying FOI model, indicating that both models performed equivalently using the seropositivity data. Therefore, using the seroprevalence study alone, we were not able to identify which scenario had the most support.”*

*Abstract:*

*“Using the reverse catalytic model, we estimated the duration of antibody persistence to be 8.33 years (4.76 - 12.50; assuming constant FOI) and 7.25 years (3.36 – 11.36; assuming time-varying FOI), which is longer than previous estimates.”*

However, by bringing in data on antibody kinetics from longitudinal data from Lupidi *et al*. and bringing in the clinically suspected cases (2012-2013 suspected clinical leptospirosis cases in Fiji), we were able to estimate the most likely timing of infection and conclude that the outbreaks seem to be more recent. This lends support for the time-varying FOI model. This highlights the challenges of trying to identify outbreaks and outbreak timing from age-structured seroprevalence data, and suggests that using an alternative approach using antibody kinetics may be more appropriate, depending on the question of interest.

We have added these points to the discussion:

*“Since antibodies can act as a marker of exposure to infection, we explored two complementary approaches to estimate the timing of infection at the population level. In the first, we used the population seroprevalence data, and allowed for a time-varying FOI, which inferred that there was endemic transmission occurring, and a large outbreak in 2013. However, there was a lot of uncertainty regarding the timing and the size of the peak, with large credible intervals, and only the binary seroprevalence data was used. In the second approach, we used the MAT antibody titres by serovar, MAT antibody titres from clinically suspected leptospirosis cases and longitudinal information on antibody decay rates from Lupidi et al . to estimate the most likely timing of infection, fully utilising the available seroprevalence data. From this, we found that most individuals included in the 2013 seroprevalence study were likely to have had a recent infection within the last two years. These results appear to correspond with what is known from surveillance data collected by the Fiji Ministry of Health, which show high numbers of cases in 2012 and 2013 [29,32]. We demonstrate that by incorporating additional sources of data, including longitudinal information on antibody kinetics, we were able to identify the timing of infection.”*

We have also updated the abstract to reflect this:

*“Using age-structured seroprevalence data alone, we were not able to distinguish between these two models. However, by bringing in additional longitudinal data on antibody kinetics we were able to estimate the most likely time of infection, lending support to the time-varying FOI model. We found that most individuals who were antibody-positive in the 2013 serosurvey were likely to have been infected within the previous two years, and this finding is consistent with surveillance data showing high numbers of cases reported in 2012 and 2013.”*

**Minor comments**

I really like the use of the Italian outbreak for estimation of time since infection. I think there are two important limitations that should be noted. Firstly, the Italians had no pre-existing immunity. In contrast the Fijians may have had pre-existing immunity, so they would have started at a higher baseline, potentially causing the estimated times since infection to be biased towards being more recent. A second important point is on the comparability of the assays used for the Italian and Fijian data. I’m not an expert in MAT assays, so can’t judge the details, but my understanding is that being a more functional assay, comparability tends to be good. No need for a major overhaul, an acknowledgement of limitations in the discussion should be sufficient.

In this study, we estimated the antibody decay rate from Lupidi et al. (non-endemic setting). However, to limit the bias between settings, we used the Fiji clinically suspected cases as our titre estimates post infection, therefore not using the Italian population as a baseline. We did this as we agree that given Italy is non-endemic, these would not have been comparable.

Regarding your point about standardisation of the MAT test, we agree and have added this point to the discussion:

*“In addition, standardisation of the MAT test is challenging, and the results may not be fully comparable across settings [45].”*

A nice point which I think could be emphasized a bit more is the consistency between the estimated duration of sero-positivity from the Italian data and the estimate from the sero-catalytic model. In general, I would put more weight on estimates of duration of immunity from longitudinal datasets than estimates from cross-sectional data.\

Thank you for your comment.

We think that it is useful to demonstrate that from cross-sectional data from a high exposure setting that we are able to estimate the duration of antibody persistence. However, it would be useful to have longitudinal datasets from high prevalence settings to validate these estimates to.

We have emphasised this in the discussion:

“*We estimated (using a linear mixed effects model) that in Lupidi et al. the time taken to reach undetectable levels was 6.57 years, which extended beyond the follow-up period, and this is in accordance with our estimate of the duration of antibody persistence from the Fiji serosurvey, suggesting that antibody decay rates are comparable across settings. However, care needs to be taken when comparing the duration of immunity in different contexts. Fiji is an endemic setting and repeated infections are more likely. These may boost antibody responses, resulting in longer persistence of measurable antibodies [6,45]. In a different, non-endemic setting, antibody persistence may be estimated to be shorter. Therefore, additional longitudinal datasets from settings with high prevalence would be useful to validate these results.*”

“In our analysis, the estimated annual attack rate for Leptospira infection in Fiji (3.15%, 2.18% - 5.16%) would suggest that there may be as many as 28,000 infections in Fiji per year, using the 2017 census”

I think uncertainty should be propagated into burden estimates.

Thank you for the suggestion, I have now added these:

*“In our analysis, the estimated annual attack rate for Leptospira infection in Fiji (3.15%, 2.18% - 5.16%) using the reverse catalytic model would suggest that there may be as many as 28,000 (19,000 – 46,000) infections in Fiji per year, using the 2017 population census.”*

In Supplementary Figure 1, the recent FOI looks very high.

This is a limitation with this approach – which also relates to some of the above points about FOI estimation given different model structures – where the time-varying FOI cannot be well estimated. From the seroprevalence alone we cannot reliably estimate the size or timing of a specific recent outbreak. Therefore, using the antibody kinetics is generally likely to be a more viable approach in this situation, as long as the kinetics can be appropriately calibrated to the specific setting of interest (e.g. Fiji rather than Italy). We have added to both the results and discussion regarding this:

Results:

*“The timing of the outbreak, when allowed to occur in the preceding five years, estimated the outbreak to be in April 2013 (95% CrI: September 2009 - December 2013; Supplementary Fig. 2), albeit with wide credible intervals and there was a lot of uncertainty regarding the height of the peak.”*

Discussion:

*“In the first, we used the population seroprevalence data, and allowed for a time-varying FOI, which inferred that there was endemic transmission occurring, and a large outbreak in 2013. However, there was a lot of uncertainty regarding the timing and the size of the peak, with large credible intervals, when only the binary seroprevalence data was used.”*

Data and code should be made open access. The GitHub link provided did not work for me.

*Apologies - you should now be able to access it.*

Reviewer #3

Methods:

The methods are well described, with sufficient data for the mathematical analysis. All applied analysis are well suited for reaching the proposed objectives.

Results:

The results are clearly presented and discussed, with implications well explained. Figures and Tables are in sufficient quality.

Conclusion:

Conclusions are well fitted to the presented results, and the author are critical about the limited quality of the data available. Results and conclusion were used to address the epidemiological situation in Fiji, and the author proposed that this model could be applied to infer force of infection and probable time of infection in different contexts.

Introduction

The number of species is outdated, since the WGS approach and ANI cutoff of 95% proposed by Vincent et al., (2019) currently recognizes 64 species divided in four subclades. Latter, Korba et al. (2021) isolated and identified additional 2 P1 (pathogenic) and 2 S1 (saprophytes) species.

Thank you for taking the time to review our manuscript. Thank you for highlighting this - we have now updated the introduction to reflect this.

*“Recent laboratory advances isolating novel species of the genus Leptospira from the environment using Next-Generation Sequencing has expanded the number of named species to 68, which includes both pathogenic and non-pathogenic species, and these have been proposed to be organised into two clades, and four subclades [10–12]. Leptospira can also be serologically classified into serogroups and serovars, and serotyping based on the heterogeneity of the surface lipopolysaccharide (LPS) has led to the identification of 25 serogroups and over 300 serovars [11,13–16]”.*

When the authors stated that ELISA protocols detect antibodies against both pathogenic and saprophytic species, to which antigens are they refereeing to? There are several recombinant proteins used as antigens that are specific to pathogenic species, as LipL32, OmpL1, Lig proteins, etc…Please, clarify or expand this statement.

Since our study was focused on MAT, detailed discussion about ELISA is outside of the scope of this paper.  We have therefore simplified this sentence to:

“The enzyme-linked immunosorbent assay (ELISA) test is most commonly used in this context as it is easier to perform and is more sensitive than the MAT test during the acute phase of the illness, but is not serogroup or serovar-specific.”

Methods

The 199 individuals who had clinical suspected leptospirosis were all positive by IgM ELISA? This excerpt is a little ambiguous.

Yes - this has now been clarified in the methods:

*“We used a serum bank of 199 individuals with clinical suspected leptospirosis and positive IgM-ELISA, collected from April 2012 to November 2013 following an outbreak in Fiji [28,32]. MATs were conducted on serum from these patients, and 66 had detectable antibodies using MAT.”*

Can the MAT titers of the 66 patients that were infected in a very close timeframe be used to correlates the MAT titers to distinct serovars to infer they intrinsic immunogenicity (more of that in the general comments).

Thank you for your comment, I have responded below (in the general comments).

Was antibody waning rate also calculated by Lupidi et al in their work?

No, Lupidi *et al.* presented data on 18 individuals, and the data from their paper was extracted in order to calculate the antibody waning rate in this paper.

General comments

As immune response is multifactorial, reflecting host age and immunocompetence, and bacterial inoculum size and serovar/species, can MAT titer be generically used to infer the probable time of infection. We observed a very heterogeneous MAT titers among patients in very similar timeframes.

Thank you for your comment. We agree that there is a lot of heterogeneity in MAT titres among infected individuals, and this is acknowledged in the discussion:

*“Finally, very little data exist on antibody profiles following infection with leptospirosis, and the available evidence demonstrates high levels of inter-individual heterogeneity. This is highlighted by Lupidi et al. [19], who reported a point source outbreak in Italy, where leptospirosis is not endemic. Each individual followed up over time in their study showed distinct antibody profiles. Despite these limitations in data quality and uncertainties in antibody dynamics, our methods were able to identify a time window in which transmission was most likely to have occurred, and which corresponds to known outbreaks in Fiji.”*

For this reason, due to the uncertainty in individual-level titre trajectories, the timing of infection was reported as an expectation at the population level, rather than the individual level infection histories. For example, suppose 100 individuals all have a 50% probability of being infected in year A or year B; at the individual level, infection timing can’t be reported with confidence, but the expectation of 50 infections in each year can be. This could have been clearer so we have added to the methods:

*“Using the MAT antibody titres from the 2013 Fiji seroprevalence survey, we estimated the timing of infection of participants. Due to uncertainty associated with individual titre estimates – and hence timings – the dynamics of infection was aggregated and reported as the population level expectation.”*

*“To reconstruct the timing of infection from the 2013 seroprevalence survey we combined the 2012 Fiji clinically suspected cases with the estimated rate at which individual responses wane by one antibody dilution titre. First, using the 2012 Fiji clinically suspected cases (n = 199), we estimated the geometric mean antibody titre from the MAT-positive cases (n = 66). We then used the MAT antibody distribution of the 2012 Fiji clinically suspected cases, combined with the antibody decay estimates from Lupidi et al. [23], to analyse the 2013 seroprevalence data. For each titre level from the 2013 seroprevalence survey, the possible initial titre levels were estimated, based on the proportions from the 2012 Fiji clinically suspected case distribution results. Using the rate of decline of antibody titres, the initial titre levels were transformed into an estimated time since infection, reconstructing the potential timing of infection at the population level. An example for one titre is shown in Fig. 2. As individuals could be seropositive for more than one serovar, two separate analyses were conducted, one where infections with different serovars were assumed to be independent events (n = 520), and one where only the highest titre was used (n = 417).”*

Also, is there any data on how MAT titers of individuals infected with serovar Pohnpei correlates to those with serovar Copenhageni or other serovars?

We have included histograms within the supplementary material, showing the distributions of titres across serovars. However, since serovar Pohnpei dominated the results, this limited our ability to perform serovar-specific analyses for other serovars given the paucity of available titre levels.

We have added to the discussion to acknowledge this:

*“A previous study describing the human serosurvey in Fiji found that there were differences in serovar distribution by age and location, suggesting that there are different risk factors of disease transmission between sub-groups [28]. For example, livestock could be more important drivers in rural areas, with rodents being more important in urban areas. However, in this setting there was one dominant serovar (serovar Pohnpei), limiting the ability to identify serovar-specific risk factors.”*

**Supplementary Figure 3.** Distribution of MAT titres by serovar for seropositive individuals. 89 individuals had titres for more than one serovar, and so are included more than once in this plot (n=520).

Could this serological survey also be done by IgM/IgG ELISA instead of MAT? Do the authors think that the seroprevalence would be higher? Higher IgM would be indicative of more recent infections.

MAT is preferable to ELISA for these types of studies because cross reactions (e.g. with arboviruses) are not uncommon with ELISA, and particularly problematic in places like Fiji where many of these diseases are common. i.e. false positives would be a problem. ELISAs should therefore be considered as a screening test, and positive results should be confirmed with MAT. Also, ELISAs are positive for a relatively short time after the acute infection, so much less useful than MAT for cross sectional serological surveys.

We hope that these amendments to the manuscript are satisfactory to address the questions and comments raised.

Kind regards,

Eleanor Rees

on behalf of all co-authors
